# Analysis of factors driving variations in neonatal parenteral nutrition over a decade: A retrospective study

**DOI:** 10.1002/ncp.70134

**Published:** 2026-05-10

**Authors:** Thanaphong Phongpreecha, Lucas W. Y. Sin, Shabnam Gaskari, Nima Aghaeepour

**Affiliations:** 1 Department of Anesthesiology, Pain and Perioperative Medicine, Stanford University, Stanford, California, USA; 2 Department of Pediatrics, Stanford University School of Medicine, Stanford, California, USA; 3 Department of Biomedical Data Science, Stanford University, Stanford, California, USA; 4 Takeoff41, Inc., Oakland, CA, USA; 5 Pediatric Pharmacy, Stanford University School of Medicine, Stanford, California, USA

**Keywords:** clinical practice variation, neonatal parenteral nutrition, NICU, nutrient composition, prescribing variation

## Abstract

**Background:**

Parenteral nutrition (PN) is essential for supporting preterm and critically ill neonates who are unable to tolerate adequate enteral feeds. However, PN composition remains highly variable due to differences in guidelines, institutional practices, prescriber discretion, and more. This lack of standardization contributes to inconsistent nutrient delivery and may affect clinical outcomes. A deeper understanding of the sources of variation is needed to inform efforts toward more consistent and evidence-aligned prescribing.

**Methods:**

This retrospective study analyzed 54,464 neonatal PN prescriptions issued between 2011 and 2021 to 5052 infants. Patient demographics, prescriber identifiers, and prescription year were linked through electronic health records. Temporal trends in nutrient composition were evaluated using linear mixed-effects models, with covariate adjustment. Dimensionality reduction with t-distributed Stochastic Neighbor Embedding (t-SNE) was applied to visualize temporal clustering of prescriptions and prescriber-level similarity in dosing patterns.

**Results:**

PN composition demonstrated clear temporal variability across the decade. Macronutrient shifts diminished after adjusting for patient demographics, indicating that changes were primarily driven by shifts in the NICU population. In contrast, most micronutrients retained significant year effects. Variance partitioning revealed that prescriber identity explained substantially more variability than prescription year for nearly all electrolytes. Guideline updates also shaped certain changes, such as Selenium, which shifted in line with a specific update rather than prescriber preference.

**Conclusion:**

Neonatal PN is influenced by multiple factors, with prescriber-level differences emerging as a dominant driver for micronutrients. These findings highlight the need for targeted standardization efforts that reduce unnecessary heterogeneity while preserving flexibility for clinical judgment.

## INTRODUCTION

Parenteral Nutrition (PN), a method of intravenous nutrition administration, is a life-saving treatment used in neonatal intensive care units (NICUs) for newborns, especially those who were born preterm.^1^ Most of these neonates either have gastrointestinal issues or immature development of their gastrointestinal tracts, disallowing the use of other feeding methods.^2^ Strict PN implies that no other forms of nutrition are provided, but in practice the term PN is often used interchangeably with TPN, even when patients are also receiving partial enteral feeds.

PN consists of multiple nutrients for growing infants, such as intravaneous lipid emulsion (ILE), amino acids, glucose, sodium, multivitamins, and trace elements such as selenium and zinc.^3^ However, there is no objective or standardized approach to dose these components.^4^ In fact, its variation is often regarded as unmatched by any other medication in clinical practice.^5^ This is due to multiple reasons ranging from different national guidelines, institutional guidelines, or even provider’s anecdotal experience.^6^ For example, most U.S. NICUs follow the guidelines from American Society for Parenteral and Enteral Nutrition (ASPEN),^7,8^ which generally recommend higher initial amino acid doses compared to their European counterparts.^7,9^ While ASPEN does not formally recommend a specific lipid injectable emulsion composition, U.S. practice has predominantly relied on pure soybean oil-based formulations,^8^ whereas European guidelines recommend composite ILEs as first-choice treatment.^9^ Similarly, there is also not an ideal fixed volume or length of PN usage either, as the total volume of PN must also account for total fluid allowance, which also varies between patients depending on their situations.^10,11^ More importantly, these guidelines are not always definitive for all ingredients, they often provide a range of recommendations, leaving the interpretation and issuing of the prescription solely to the provider.^7^ The guidelines could also change over the years, with different adoption rates by different institutions. All of these lead to the inconsistent dosage of PN prescription within and across hospitals.

The variation in the length, initiation time, volume, and nutritional values of PN could have significant effects on patients’ outcomes.^12,13^ This can range from short-term effects such as hyperglycemia and hyponatremia to associations with more serious conditions, including necrotizing enterocolitis (NEC), bronchopulmonary dysplasia (BPD), sepsis, and hepatic dysfunction.^8,14–18^ Despite this, there is yet a large clinical trial to support the best PN composition, potentially due to complex and highly diverse needs of neonatal population.^19^ Some agencies aim to tackle this problem with some level of standardization and structured protocols, such as based on weight from the Australasian Society for Parenteral and Enteral Nutrition (AuSPEN).^20^ While some studies have shown that this approach is non-inferior to individualized practice like in the U.S.,^21^ others have shown contradicting outcome results.^22^

To address variation in PN prescribing, we must first understand the primary source of each type of variation, which may differ across individual PN components. In this study, we leverage a decade of PN data linked to patient demographics and prescribers, allowing us to disentangle multiple contributing factors and identify potential drivers of variation for each ingredient. By clarifying these patterns, we can better inform standardization efforts and potentially guide more consistent, evidence-aligned prescribing practices.^23^

## METHODS

### Study design and setting

Cohort data were aggregated from the electronic health records (EHRs) at Stanford Health Care, an academic medical center in the United States. The NICU includes a 40-bed Level IV unit with a specialized 7-bed unit dedicated to infants born under 29 weeks’ gestation, advanced ECMO capability, and specialized neonatal surgery, as well as a 16-bed Level II unit. An average of approximately 15 PN orders were placed per day. The mean birth weight and gestational age of the cohort were 2.37 kg and 35 weeks, respectively. PN initiation followed a weight-based fluid protocol: 100 mL/kg/d for infants <1000 g, 80–100 mL/kg/d for 1000–1500 g, and 60–80 mL/kg/d for >1500 g, with total fluid goals of 150–160 mL/kg/d (micro-preemies <800 g may require ≥200 mL/kg/d). Enteral feeds are typically introduced throughout the 1st week of life, with PN volume adjusted accordingly as the total fluid goal is achieved. The transition from PN to full enteral nutrition varies by individual clinical course.

The linkage of the datasets enabled the integration of nutritional data, phenotypic traits and prescriber. All EHR data were mapped to the Observational Medical Outcomes Partnership Common Data Model v.5.3.1. The study was approved by the Institutional Review Board of Stanford University (reference no. 39225) with informed consent waived. We first identified a cohort of 6991 neonatal/pediatric patients in NICU/pediatric intensive care units who received a total of 113,773 PN orders recorded between January 2011 and January 2022. These PN data represent best practice in the field and come as close as possible to representing the gold standard given the knowledge currently available to the field. All PN orders were developed by a multidisciplinary healthcare team. Each order is placed by a neonatologist, house staff resident or neonatal nurse practitioner, and is then checked by a NICU clinical pharmacist and a NICU dietitian. They also use custom modules embedded in the EHR for automated calculations, including calcium-phosphate solubility checks, osmolarity calculations, alerting for values outside acceptable ranges, and more. Of the 6991 patients identified, 5052 were retained as they received PN specifically within NICU (no Pediatric ICU or Cardiovascular ICU included) by the end of 2021, which resulted in 54,464 PN orders. The year 2022 was not included as we only have its January data and hence would not accurately represent the year. Sex and gender were not considered in the design of the study, as this is a retrospective study of all available patients that fit the inclusion criteria. These data include patients receiving both total and supplemental parenteral nutrition (Table S1).

### Statistical analysis

To assess how prescription year influenced the nutrient content of neonatal PN, the analysis applied a series of linear mixed-effects regression models,^24^ treating each nutrient as a separate outcome. Multiple PN prescriptions often occur within the same hospital stay, so patient ID served as a random intercept to capture individual baseline differences and account for within-patient correlation. Prescription year was specified as the primary fixed-effect predictor to quantify temporal changes in nutrient provision. Gestational age at birth, birth weight, sex, and postnatal day at the time of prescription were included as additional fixed effects to control for clinical and developmental factors known to influence PN composition and to avoid confounding by changing patient characteristics over time. Models were fitted using the MixedLM implementation in the statsmodels framework,^25^ which supports maximum likelihood and restricted maximum likelihood estimation. Model diagnostics involved inspection of residual distributions and verification of model convergence. The fixed-effect coefficient for prescription year was interpreted as the estimated annual change in the prescribed amount of each nutrient.

To evaluate how much variability in nutrient prescription could be attributed to prescribing behavior versus calendar year, an additional set of linear mixed-effects models was constructed for each PN nutrient. In these models, the nutrient served as the outcome, and gestational age, birth weight, sex, and postnatal day were included as fixed-effect covariates to account for clinical factors influencing nutrient requirements. Two versions of the model were estimated: one specifying prescriber as the random intercept, and another specifying year as the random intercept. These random-effect terms allowed the models to partition the variance in nutrient prescriptions attributable to differences among individual prescribers or across calendar years, respectively. Each model was also fitted using the MixedLM implementation in the statsmodels framework. The proportion of total variance explained by the random effect was calculated as the random-effect variance divided by the sum of random-effect and residual variances.

### t-SNE visualization

The 16-dimension PN prescription data was reduced to 2D for visualization using t-distributed Stochastic Neighbor Embedding (t-SNE).^26^ For prescription-level visualization, we use all prescription data in the dataset. For the prescriber correlation network, the data was aggregated at the prescriber level using the mean PN dosage value before feeding into t-SNE algorithm to obtain the 2D coordinate. Each of these node coordinates represent one prescriber. The edge between two nodes was drawn if the *P*-value correlation between the two is less than 0.0001.

## RESULTS

### PN composition is growing in importance as patients on long-term PN increase

This study analyzed neonatal PN prescription data from 2011 to 2021, encompassing 54,464 prescriptions issued to 5052 unique infants. During this period, 652 prescribers contributed orders, each potentially using their own composition patterns. The dataset included a broad range of nutrient components commonly used in neonatal parenteral nutrition: amino acid dose (g/kg), dextrose as measured by glucose infusion rate (GIR; mg/kg/min), acetate (mEq/kg), calcium (mg of calcium gluconate/kg), copper (mcg/kg), famotidine (mg/kg), levocarnitine (mg/kg), magnesium (mEq/kg), multivitamins (mL), phosphate (mEq/kg), potassium (mEq/kg), selenium (mcg/kg), sodium (mEq/kg), zinc (mcg/kg), ILE or fat dose (g/kg), chloride (mEq/kg), and the total PN volume dose (mL/kg). All PN ingredients are listed in Table S2. Briefly, birth weights in the cohort ranged from 0.34 to 5.57 kg, spanning the standard clinical categories of normal birth weight (≥2500 g), low birth weight (<2500 g), very low birth weight (<1500 g), and extremely low birth weight (<1000 g).

**FIGURE 1:**
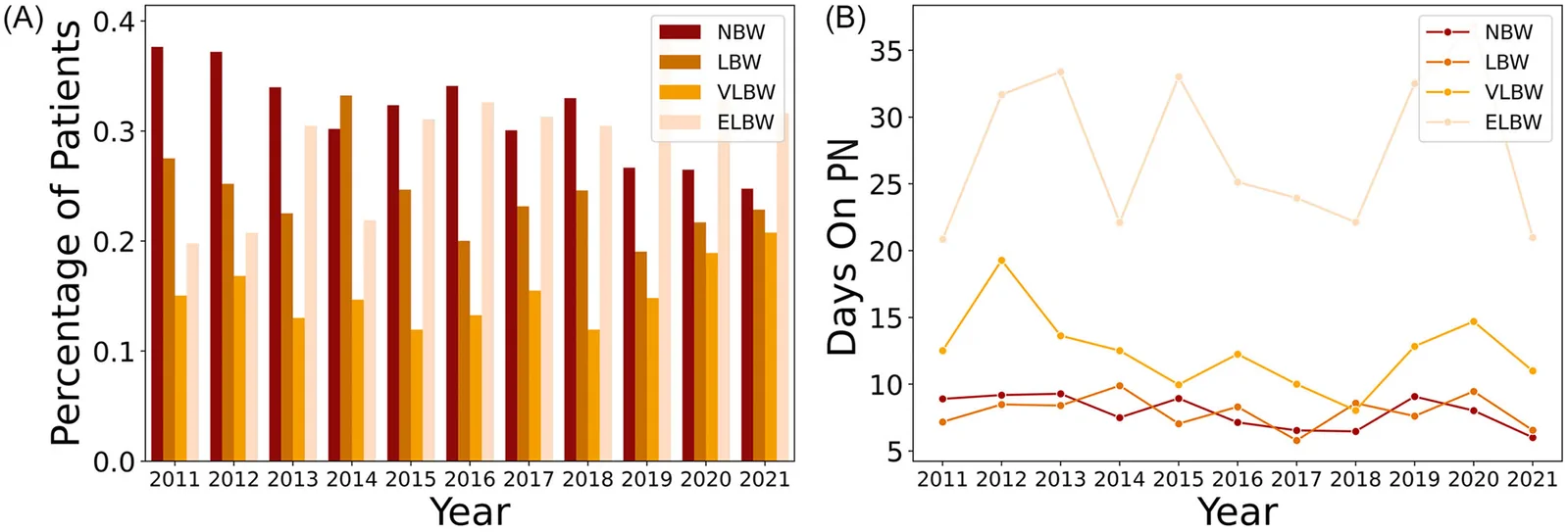
Temporal shifts in neonatal birth-weight distribution indicate higher preterm population. (A) Annual proportion of NICU patients receiving PN, stratified by birth-weight category. (B) Average number of days on PN per year for each birth-weight group remains the same.

As the PN composition over the years could be affected by the change in population, as well as the duration of PN therapy, we first investigated the trend of these factors for the study period. In our data, we observe a clear trend of lower number of term newborns over the years, particularly there was a sharp rise in all other preterm categories in recent years (Figure 1A). On the other hand, within each of these birth weight populations, there was no apparent trend in the number of days on PN over the year (Figure 1B). These suggest that the PN composition could be mostly affected by the shift in NICU population, and less so by changes in PN duration.

### Association between year of prescriptions and PN compositions

**FIGURE 2:**
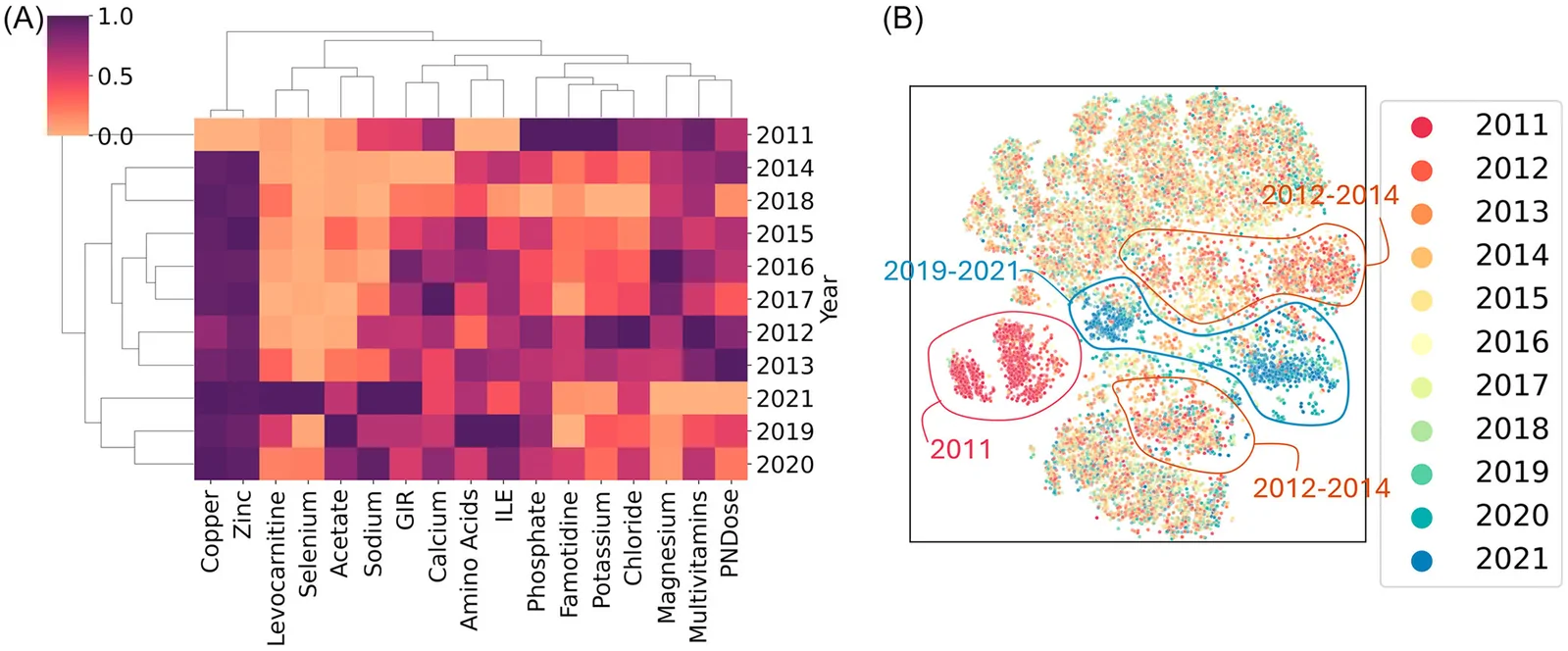
Patterns in PN composition reveal year-dependent nutrient profiles. (A) Heatmap of annual mean nutrient values, standardized across components, with hierarchical clustering applied to both nutrients and years. (B) Two-dimensional t-SNE embedding of all individual PN prescriptions, colored by year.

The composition of PN, and to a lesser extent its volume, exhibited variable changes across the decade of prescription records. When these changes were clustered by year, smaller differences were observed between consecutive or nearby years compared to larger gaps (Figure 2A). For example, prescriptions from 2011 to 2018 typically contained higher magnesium, multivitamins, and total volume than those from 2019 onward, whereas selenium concentrations were lower in the earlier period than from 2019 onward. These shifts could arise from a multitude of factors that will be investigated further. A high-level overview is provided by the t-SNE plot of prescriptions colored by year, which reveals that prescriptions from 2019 onward form the tightest cluster, as indicated by the proximity of their points (Figure 2B). Similarly, 2011–2013 prescriptions cluster tightly together, followed by a somewhat looser mixed group from 2013 to 2014. Prescriptions from intervening years are less distinctly separated. Together, these analyses demonstrate clear temporal changes in PN prescribing practices and suggest the presence of underlying trends.

**FIGURE 3:**
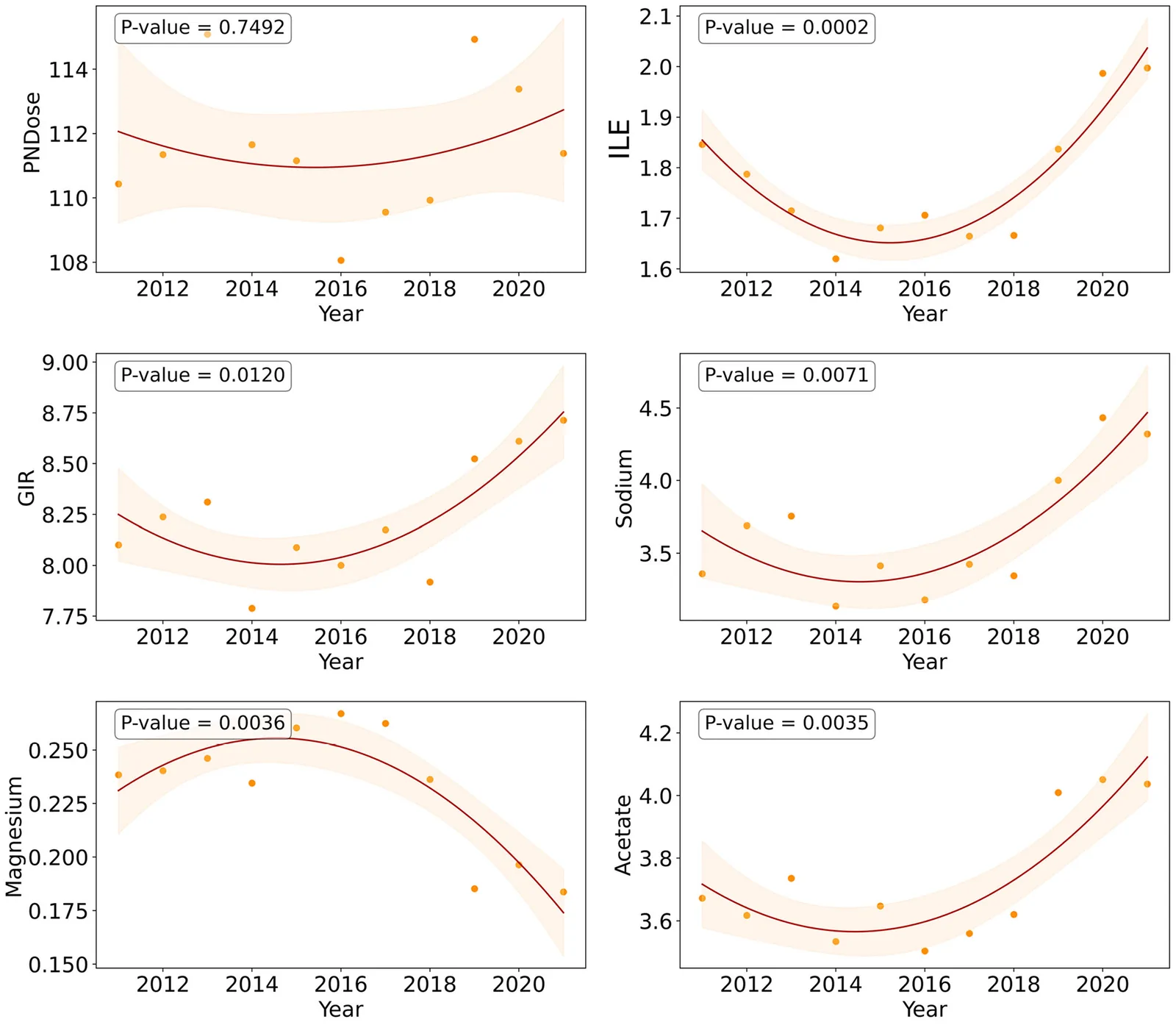
PN components display nonlinear temporal trajectories across the study period. Annual mean values for selected nutrients (total PN volume dose, ILE dose, glucose infusion rate, sodium, magnesium, and acetate) are shown with a second-order polynomial fit and 95% confidence interval. GIR, glucose infusion rate.

Examination of individual PN components that displayed substantial temporal variation revealed clear trends over the study period (Figure 3). ILE, GIR, and most electrolytes gradually declined from 2014 to 2018 relative to the early 2010s, before rebounding to levels that exceeded early-decade values from 2019 onward. In contrast, magnesium followed an opposite trajectory: highest in the early 2010s, slightly more elevated through the mid-2010s, then dropping sharply in recent years. All reported trends were statistically significant (*P* < 0.05). Notably, PN volume (mL/kg/d) showed no comparable pattern and lacked statistical significance. This dissociation is meaningful, as higher PN volume typically correlates with greater dosage of macronutrients and electrolytes at the individual patient level (reflecting heavier reliance on nutrient delivery by parenteral as opposed to enteral nutrition). The absence of a strong temporal trend in PN volume, despite robust changes in nutrient density, indicates that prescribing preferences for nutrient concentration within a given PN volume have changed over time.

### Identifying potential sources of changes in PN composition

The temporal changes in PN composition may stem from, or be confounded by, several factors, including evolving institutional protocols, updated ASPEN guidelines, shifts in patient population or NICU admission criteria, turnover in prescribing providers, drug shortages, or interactions among these elements. In this section, we systematically evaluate the contributions of selected factors to isolate their relative influence on observed trends.

**FIGURE 4:**
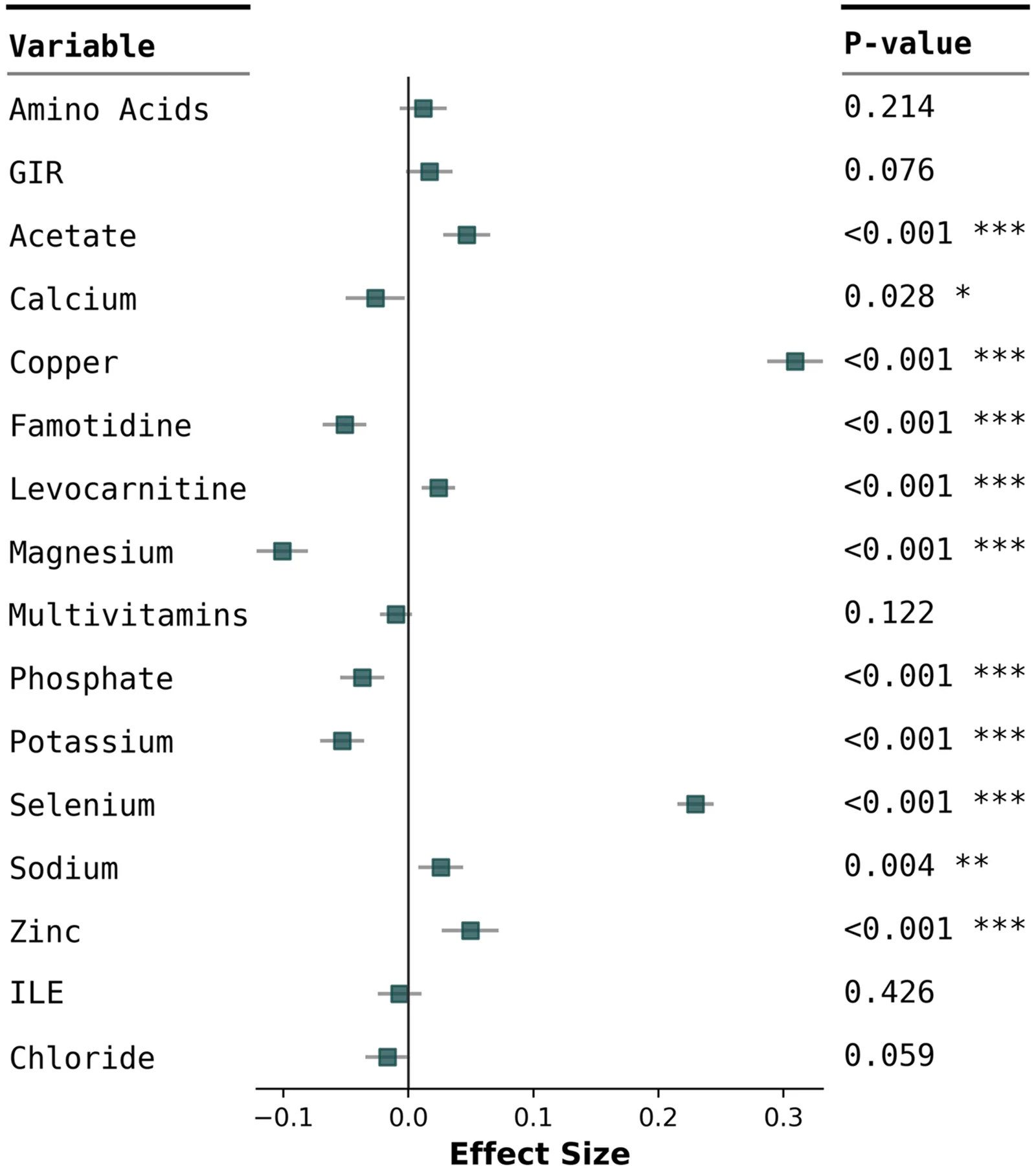
Year effects reveal selective temporal shifts in micronutrients, with minimal changes in macronutrients after demographic adjustment. Forest plot of estimated fixed-effect coefficients for prescription year from linear mixed-effects models fitted separately for each PN component and covariates adjusted by demographic. GIR, glucose infusion rate; ILE, intravaneous lipid emulsion.

To disentangle the contributions of evolving prescribing practices from shifts in patient demographics, we fitted a linear mixed-effects model for each PN component, with year as the primary predictor and postnatal age (days of life), birth weight, and gestational age included as fixed-effect covariates. Patient identifier was incorporated as a random intercept to account for repeated measurements within individuals. After adjustment, the temporal effect of year became non-significant for all macronutrients (ILE, glucose infusion rate, and amino acids), indicating that observed changes in these components were largely attributable to variations in patient population, such as differences in case mix, admission criteria, or duration of NICU stay (Figure 4). It should be noted that this reduced significance could partly result from the model assuming a strictly linear trend, whereas the previous section showed slightly polynomial patterns (Figure 3). In contrast, most electrolytes and trace elements retained highly significant year effects (*P* < 0.05), suggesting that their temporal trends could not be explained by population change alone. Note that the effect size in some trace minerals was much larger because of their almost binomial distribution, e.g., either 0 or 3 mcg/kg for selenium. These results strongly suggest that macronutrient delivery has been more sensitive to evolving patient characteristics, whereas micronutrient composition has undergone deliberate, less population-dependent modification over time.

**FIGURE 5:**
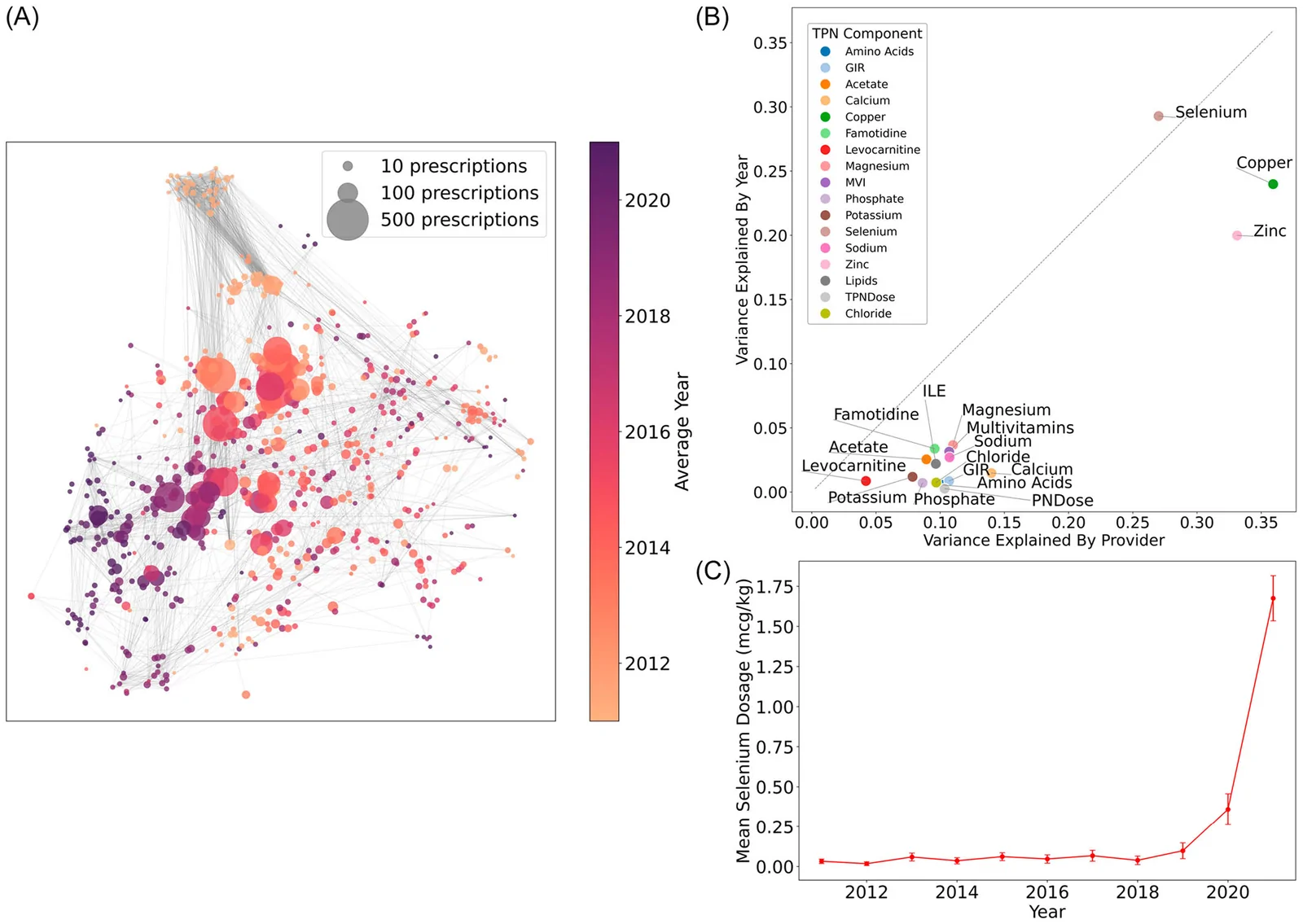
Prescriber-level differences are a dominant source of variation. (A) Visualization of prescriber-level PN. Each node represents a prescriber, edges represent significant pairwise correlations (*P* < 0.0001). (B) Variance explained by prescriber versus year. (C) Selenium across years reflects guideline changes. GIR, glucose infusion rate; ILE, intravaneous lipid emulsion.

Prescriber preference could play a huge role in changes of electrolyte dosages throughout the years. Throughout the decademore than, 652 prescribers contributed orders, many of whom ordered a small-to-medium number of PNs (Figure 5A). This likely stems from institutional practice where there are mainly three PN prescribing teams in the NICUs: one is a monthly rotating resident team, which could contribute to the higher number of prescribers, and the other two are a mix of hospitalists and neonatal nurse practitioners. Note that all teams work with dietitians and clinical pharmacists. Similar to previous prescription plots using t-SNE (Figure 2B), where prescriptions in early 2010s and those from 2019 onward were tightly grouped together, prescribers nodes also exhibited resemble trends although with slightly more spread out (Figure 5A). This implies a critical likelihood that the temporal changes were confounded, or caused, by changes in prescribers.

To determine if the changes in PN composition over the years could stem from changes in prescribers, mixed-effects models were used with either year or prescriber as the random effect. Weight, age (days of life), and gestational age were included as fixed effects. The variance explained by each random effect was calculated, and for all nutrients except selenium, the variance explained by the prescriber was higher than by year (Figure 5B). For example, the variance explained for sodium was almost 3x higher by prescribers. Although it should be noted that guideline changes still play some role, and selenium is the best example, as its variance explained is not higher by prescriber. The selenium trend shows a sharp increase from 2019 onward (Figure 5C), which corresponds to institutional adoption of selenium from a default of 0 to 3 mcg/kg in most neonatal cases. Together, these results highly suggest that changes in prescribers are the stronger predictor of variation in PN components. And because prescribers changed across the years, the observed PN changes across the years were likely driven by prescriber-level differences.

## DISCUSSION

This study highlights the critical role, yet highly variable, of parenteral nutrition (PN) in supporting neonates in the NICUs whose immature gastrointestinal systems often necessitate prolonged parenteral support. Despite the life-saving nature of PN, its composition lacks standardization, with prescriptions varying widely due to a variety of reasons, such as different guidelines, potentially broad guideline ranges, and prescriber discretion. Using a decade (2011–2021) of neonatal PN prescriptions from a large academic NICU, comprising over 54,000 orders from 5052 infants, this analysis revealed clear temporal shifts in PN composition, whether it be macronutrients, electrolytes, or trace elements. These changes were not uniform: macronutrient trends largely disappeared after adjusting for patient demographics, whereas electrolytes and most trace elements retained strong year effects, with prescriber identity explaining far greater variance than calendar year alone.

These findings are consistent with clinical context in neonatal nutrition. Macronutrient delivery (amino acids, glucose, ILE) is guided by relatively strict, weight- and age-based protocols that have been refined over decades. In contrast, electrolyte dosing has more possible/flexible range by most guidelines, making it remains heavily influenced by individual prescriber judgment and anecdotal experience, as reflected in numerous reports of wide inter-provider variability even within the same unit.^27,28^ Meanwhile, trace elements often follow more institutional defaults, explaining why guideline-driven changes dominate their trends while prescriber effects are minimal.

Throughout the decade that data were collected, there were substantial changes in preterm infant treatment that could impact outcome rates. In 2013, O_2_ saturation goals were changed from 88%–92% to 90%–95% (based on the SUPPORT trial), and critical CHD screening with pulse oximetry was introduced. In 2014, cooling on transport for HIE was adopted. In 2015, the brain care bundle, small baby unit, and standardized post-extubation respiratory support based on weight were introduced. In 2016, standardized rapid sequence intubation and premedication were established. In 2017, the PREMILOC protocol for low-dose hydrocortisone in extremely preterm infants began (ended in 2019), alongside a shift to stop PN and remove central lines once feeds reached 100 mL/kg/d. In 2018, PDA catheter-based closure with the Piccolo device was introduced. Other changes, such as using minimally invasive surfactant therapy (LISA/MIST) for surfactant administration, faster feeding pathways after NEC diagnosis, and higher FiO_2_ tolerance with bubble CPAP before intubation were not implemented until 2023, which is beyond the range of our data. Notably, there have been no great changes in the way that TPN has been prescribed or administered over the study period apart from recommendation changes from ASPEN guidelines.

Limitations of this work include its single-center design, which may not fully generalize to other institutions with different protocols, staffing models, or patient populations. Additionally, the absence of concurrent enteral feeding data prevents full assessment of total nutrient intake and parenteral-enteral balance, a key determinant of PN composition. Despite these constraints, the marked prescriber-driven variation uncovered here underscores needs to reduce unintended heterogeneity in PN prescribing. A promising path forward could be in data-driven, evidence-based standardization. Future work should leverage large institutional datasets and artificial intelligence to help define safe, effective, and consistent PN templates that minimize anecdotal variation while preserving flexibility for individual patient needs. Such an approach could enhance reliability, reduce complications, and ultimately improve outcomes in this vulnerable population.

## CONCLUSION

This study demonstrates that the observed changes in neonatal PN composition across years were driven by multiple factors rather than a uniform annual trend. Shifts in patient population accounted for most macronutrient variations, as their temporal trends became non-significant after demographic adjustment. In contrast, electrolytes and most trace elements showed strong prescriber-dependent effects, reflecting persistent heterogeneity in individual practice, whereas certain trace minerals were also primarily influenced by discrete guideline updates at some years. These findings are clinically significant, given that inconsistent nutrient delivery in sick infants can heighten risks of metabolic complications and adversely affect neurodevelopmental outcomes. To ensure safety and equity, greater standardization of PN formulations, ideally guided by a data-driven approach, represents a critical next step in optimizing neonatal nutrition.

## Supplementary Material

Supplementary Materials

Additional supporting information can be found online in the Supporting Information section at the end of this article.
