# Supplementary material for: Analysis of factors driving variations in neonatal parenteral nutrition over a decade: A retrospective study
Source: Nutr Clin Pract. Author manuscript; Available in PMC 2026 Aug 4. (PMC13436136; doi:10.1002/ncp.70134)
Supplement: Supplementary Materials [file NIHMS2194027-supplement-Supplementary_Materials.docx]

Table S1. Cohort Characteristics

| **Patient Characteristics** | **Count/Mean±SD** | **Percentage** |
| --- | --- | --- |
| Number of Patients | 5052 |  |
| Days on PN | 10.78±17.03 |  |
| Days since birth | 28.17±39.29 |  |
| Daily weight (kgs) | 2.32±1.23 |  |
|  |  |  |
| **Birth weight (kgs)** | 2.27±0.97 |  |
| <1 kgs | 566 | 11.20 |
| 1-2.5 kgs | 2370 | 46.91 |
| >2.5 kgs | 2116 | 41.88 |
|  |  |  |
| **Gestational age (weeks)** | 34.26±4.47 |  |
| <28 weeks | 568 | 11.24 |
| 28-32 weeks | 806 | 15.95 |
| 32-34 weeks | 816 | 16.15 |
| 34-37 weeks | 1015 | 20.09 |
| >=37 weeks | 1847 | 36.56 |
|  |  |  |
| **Sex** |  |  |
| Male | 2859 | 56.59 |
| Female | 2192 | 43.39 |
| Unknown | 1 | 0.02 |
|  |  |  |
| **Race** |  |  |
| White | 2412 | 47.74 |
| Asian | 1076 | 21.30 |
| Black or African American | 146 | 2.89 |
| Native Hawaiian or Other Pacific Islander | 122 | 2.41 |
| American Indian or Alaska Native | 19 | 0.38 |
| Unknown | 1277 | 25.28 |
|  |  |  |
| **Feeding Line/Protocol** |  |  |
| PN (no enteral feed) | 32,844 | 60.30 |
| Supplemental PN (some enteral feed) | 21,620 | 39.70 |
| Central Line | 45756 | 84.01 |
| Peripheral Line | 8708 | 15.99 |

**Table S2.** Possible ingredients used in neonatal PN formulations. Not all ingredients are included in every PN order; components are selected and dosed based on individual patient needs.

| **Category** | **Product** |
| --- | --- |
| Amino acids | Trophamine 10%, Travasol 10% |
| Lipid injectable emulsions | Intralipid 20% (SO), SMOFlipid 20% (SO/MCT/OO/FO), Omegaven 10% (FO) |
| Dextrose | Dextrose 70% in Water |
| Electrolytes | Sodium chloride, Sodium acetate, Sodium phosphate, Potassium chloride, Potassium acetate, Potassium phosphate, Magnesium sulfate, Calcium gluconate |
| Trace elements | Zinc chloride, Cupric chloride, Selenious acid |
| Vitamins | Pediatric multivitamins |
| Other additives | L-Cysteine, Levocarnitine, Famotidine, Heparin |
| Sterile diluent | Sterile Water for Injection |

*Abbreviations: SO, soybean oil; MCT, medium-chain triglycerides; OO, olive oil; FO, fish oil.*
